# Nutrition as a Key Modifiable Factor for Periodontitis and Main Chronic Diseases

**DOI:** 10.3390/jcm10020197

**Published:** 2021-01-07

**Authors:** Prescilla Martinon, Laurie Fraticelli, Agnes Giboreau, Claude Dussart, Denis Bourgeois, Florence Carrouel

**Affiliations:** 1 Laboratory “Systemic Health Care”, University of Lyon, University Claude Bernard Lyon 1, EA4129, 69008 Lyon, France; prescilla.martinon@univ-lyon1.fr (P.M.); laurie.fraticelli@univ-lyon1.fr (L.F.); claude.dussart@univ-lyon1.fr (C.D.); denis.bourgeois@univ-lyon1.fr (D.B.); 2 Institute Paul Bocuse Research Center, 69130 Ecully, France; agnes.giboreau@institutpaulbocuse.com

**Keywords:** chronic diseases, periodontal diseases, prevention, nutrition, diet, nutriment, risk factor

## Abstract

Nutrition is recognized as an essential component in the prevention of a number of chronic diseases, including periodontal disease. Based on these considerations, a better understanding is required regarding how the diet, and more particularly the intake of macronutrients and micronutrients, could impact the potential relationship between nutrition and periodontal diseases, periodontal diseases and chronic diseases, nutrition and chronic diseases. To overcome this complexity, an up-to-date literature review on the nutriments related to periodontal and chronic diseases was performed. High-sugar, high-saturated fat, low-polyols, low-fiber and low-polyunsaturated-fat intake causes an increased risk of periodontal diseases. This pattern of nutrients is classically found in the Western diet, which is considered as an ‘unhealthy’ diet that causes cardiovascular diseases, diabetes and cancers. Conversely, low-sugar, high-fiber and high-omega-6-to-omega-3 fatty acid ratio intake reduces the risk of periodontal diseases. The Mediterranean, DASH, vegetarian and Okinawa diets that correspond to these nutritional intakes are considered as ‘healthy’ diets, reducing this risk of cardiovascular diseases, diabetes and cancers. The role of micronutrients, such as vitamin D, E, K and magnesium, remains unclear, while others, such as vitamin A, B, C, calcium, zinc and polyphenols have been shown to prevent PDs. Some evidence suggests that probiotics and prebiotics could promote periodontal health. Periodontal and chronic diseases share, with a time delay, nutrition as a risk factor. Thus, any change in periodontal health should be considered as a warning signal to control the dietary quality of patients and thus reduce the risk of developing chronic diseases later on.

## 1. Introduction

Nutrition is a critical component of lifelong health and development [1]. Better nutrition improves the health of infants, children and adults, reinforces the immune system, promotes safer pregnancy and childbirth, contributes to longevity and reduces the risk of non-communicable diseases [2]. Thus, nutrition and health are strongly connected. Today, the world is facing a double burden of malnutrition, including both undernutrition and overnutrition. Both forms of malnutrition are a major challenge to human health [3]. Increasing rates of overweight and obesity around the world are accompanied by soaring rates of chronic diseases (CDs) such as cardiovascular diseases (CVDs), diabetes and cancer [4,5]. It is, however, important not to link malnutrition only with obesity and major CDs. Periodontal disease (PD) is a noncommunicable disease with a 45–50% global prevalence, with 11% of the global population suffering from a severe form, which makes it the sixth most common disease [6]. The high prevalence of PDs among teenagers, adults and seniors is a public health problem [7]. PDs are oral multifactorial immunological, inflammatory diseases induced by oral microorganisms.

Nutrient diversity has a significant impact on periodontal conditions for all ages [8]. Balanced nutrition plays a major role in maintaining the symbiosis between oral microbiota and periodontal health [9]. Although many macro- and micronutrients enter the gastrointestinal tract through saliva, the chewing process is an essential element of the nutrient acquisition process. It involves the integrity of the periodontal tissue, influenced by several factors, such as tobacco, oral hygiene, epigenetic and genetic factors, nutrition and systemic health.

Moreover, an association exists between PDs and major CDs, such as CVD, diabetes, and stroke, as well as lung disease and rheumatoid arthritis [10,11]. Individuals with PD have a 19% greater risk of suffering from CVDs than healthy individuals and this risk reaches 44% in individuals 65 years of age and older [7]. It is also suggested that there is a bidirectional long-term impact of periodontitis and the main non communicable diseases [12,13].

Nutrition, PDs and the major CDs thus form an interdependent relationship throughout life. The so-called ‘common risk factors approach’, which is well accepted in public health, combines the prevention of CDs with the control of PDs, primarily by addressing a diversity of modifiable factors such as dietary risk factors e.g., micro- and macronutrients, sugar consumption, and alcohol abuse [14]. This approach suggests that a combination of interventions on the lowest common denominator, i.e., nutrition, could improve health outcomes. To the best of our knowledge, the potential three-directional interactions between nutrition, PDs and CDs have never been analyzed.

The aim of this review was to collect available scientific data on the potential relationship between nutrition/PD, nutrition/CDs, and PDs/CDs. The objective is to assess if a combined nutrition strategy could contribute to reducing CD risk through a reduction in the severity and incidence of PDs.

## 2. Materials and Methods

A review based on the principles of a scoping review was conducted to provide an overview of the available research evidence [15,16]. Unlike systematic reviews that involve a comprehensive search for studies with specific designs, the scoping review is particularly useful when the information on a topic is complex and diverse [17]. This method allows the inclusion of all study designs using the following steps: (1) identification of a clear research objective and search strategies, (2) selection of relevant publications, (3) categorization of the publications, (4) extraction of data, and (5) summarizing, analyzing and reporting the results.

### 2.1. Selection Process of Publications

**Figure 1:**
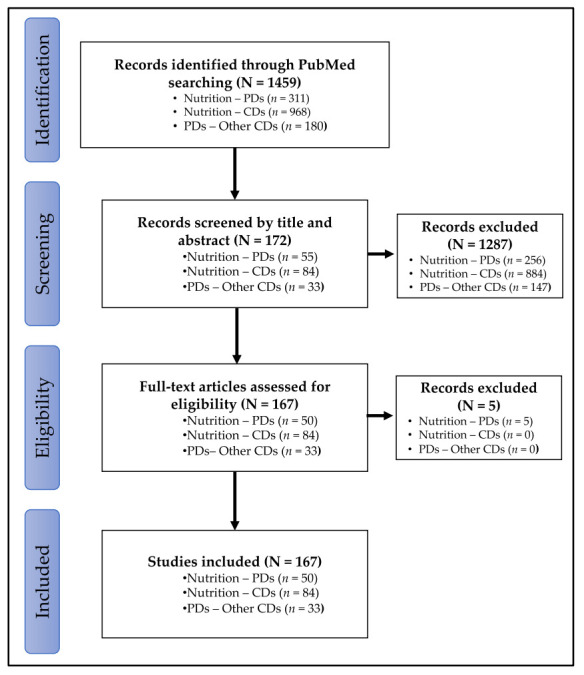
Flowchart of study selection process. CDs: chronic diseases; PD: periodontal diseases.

The general characteristics of scoping reviews included in this study are reported in Figure 1.

#### 2.1.1. Identification Strategy

Publications in English from September 2010 to September 2020 were identified in PubMed using the following search terms:

- Nutrition-PDs associations: “nutrition” (All Fields) OR “diet” (All fields) AND “periodontal disease” (All Fields) AND “humans” (MeSH Terms);
- Nutrition-CDs associations: “nutrition” (All Fields) OR “diet” (All fields) AND “chronic disease” (MeSH Terms) AND “humans” (MeSH Terms) AND (“cardiovascular disease” (All fields) OR “diabetes” (All fields) OR “cancer” (All fields);
- PDs-other CDs: “periodontal disease” (All Fields) AND “humans” (MeSH Terms) AND “chronic disease”.

Congress abstracts or commentaries were excluded. A total of 1451 publications were identified, with 303 studies for nutrition-PDs, 968 studies for nutrition-CDs and 180 for PDs-other CDs (Figure 1).

#### 2.1.2. Screening and Eligibility of Publications

Selection was refined by reading the titles and abstracts of the 1459 studies. Thus, 1287 publications were excluded because they concerned specific populations (pregnant women, elderly), behavioral aspects (mastication, hygiene) or beverages (alcohol, coffee, tea or, sodas). The full texts of the remaining 172 publications were then screened. Five publications out the scope were excluded, whereas the 167 remaining publications were included (Figure 1).

### 2.2. Determination of the Link between Nutrition, Periodontal Diseases and Other Chronic Diseases

#### 2.2.1. Classification of Publications According to the Level of Evidence

**Figure 2:**
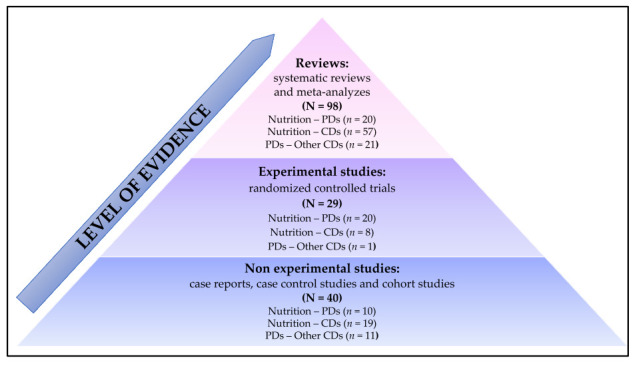
Classification of publications according to the level of evidence. CDs: chronic diseases; PD: periodontal diseases.

From the 167 publications, the studies were classified by level of evidence [18]: non-experimental studies (i.e., observational studies: case reports, case control studies and cohort studies), experimental studies (i.e., randomized controlled trials) and reviews (i.e., systemic reviews and meta-analyzes) (Figure 2).

#### 2.2.2. Data Extraction from the Included Relevant Studies and Classification of Publications According to the GRADE Process and Summarizing of the Results

For each publication in each level of evidence and category (nutrition-PDs, nutrition-CDs or PDs-other CDs), the size of the study population, the study design, objectives, results and conclusions obtained were analyzed and coded using the GRADE process. The GRADE process was used to assess the quality of the studies [19,20]. The publications were graded independently by two assessors using the following levels:

- High: The true effect lies close to that of the estimate of the effect;
- Moderate: The true effect is likely to be close to the estimate of the effect, but there is a possibility that it is substantially different;
- Low: The true effect may be substantially different from the estimate of the effect;
- Very low: The true effect is likely to be substantially different from the estimate of effect.

Any discordant grading was discussed by the assessors until a consensus was reached.

#### 2.2.3. Summarizing of the Results

**Figure 3:**
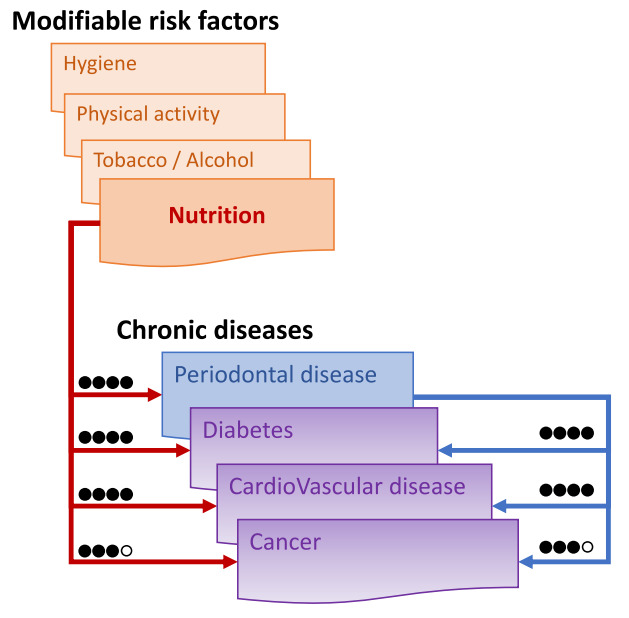
Assessment of the links between nutrition, periodontitis and other main chronic diseases based on the Grade process: high (●●●●) when the true effect lies close to that of the estimate of the effect, moderate (●●●○) when likely to be close to the estimate of the effect, but there is a possibility that it is substantially different, low (●●○○) when may be substantially different from the estimate of the effect, and very low (●○○○) when likely to be substantially different from the estimate of effect.

The assessors then determined the resulting grade for nutrition-PDs, nutrition-CDs and PDs-other CDs by combining the results obtained from the level of evidence and from the GRADE process. The following codifications were used: high (●●●●), moderate (●●●○), low (●●○○) and very low (●○○○). Any discordant grading was discussed by the assessors until a consensus was reached. The results are summarized in Figure 3.

## 3. Link between Nutrition, Periodontitis and Other Main Chronic Diseases: Research Outcomes

### 3.1. Nutrition as Risk Factor of Periodontal Diseases

#### 3.1.1. Impact of Macronutriments on Periodontal Diseases

Macronutrients consist of carbohydrates, proteins and fats. These are the nutrients from the food supply that provide energy to the body and ensure the proper functioning of its vital functions.

Carbohydrates are composed of sugars, starches and fibers that have different effects on PDs. While sugar and starches are sources of glucose, fibers are a nondigestible form of carbohydrates [21]. The main sources of carbohydrates are fruits, vegetables, whole grains, milk, and milk products. While grains and certain vegetables (potatoes and corn) are rich in starch, sweet potatoes are rich in sucrose. Dark-green vegetables and fruits are sources of sugars and dietary fiber [21]. Based on the American Dental Association guideline goal, the 2010 dietary guidelines for Americans, and carbohydrate consumption, Feinman et al. (2015) have defined a high-carbohydrate diet as a diet in which carbohydrates make up more than 45% of total energy intake and a low-carbohydrate diet as a diet in which carbohydrates make up less than 26% of total energy intake [22]. In the cohort of the National Health And Nutrition Examination Survey (NHANES) data, a high consumption of added sugar was associated with a higher PD prevalence ratio of 1.42 (95% CI, 1.08–1.85) [23]. The risk of gingival infection decreased to approximately half when the consumption of carbohydrates was restricted in a four-week diet [24]. Depending on the nature of the carbohydrates consumed, the effect on PD will be different. An excessive consumption of sugar or refined carbohydrates promoted microbiota dysbiosis that induced an inflammatory reaction and caused the apparition of PDs [25,26] (Table 1). Moreover, glucose acts on periodontal ligament cells by promoting their apoptosis and inhibiting their proliferation [27]. Conversely, polyols and fibers demonstrated a protective effect on the prevention of PDs (Table 1). Xylitol produced by the hydrogenation of xylose sugar had an antibacterial effect against *Porphyromonas gingivalis* (*P. gingivalis*) and *Aggregatibacter actinomycetemcomitans* (*A. actinomycetemcomitans*), two periodontal bacteria [28,29,30]. The results from a double-blind, randomized controlled trial comparing the effect of sugar-free chewing gum sweetened with xylitol or maltitol with a chewing gum base or no gum on gingivitis and plaque scores coupled with brushing or not showed that a reduction in sugar consumption, associated with scaling and root planing, and the use of gums containing xylitol or maltitol, can improve periodontal health [29]. The difference observed could be due to the fact that contrary to glucose, polyols are not metabolized by most oral bacteria [31]. Dietary fiber intake is inversely correlated to PD, as demonstrated in the analysis of the results of the cohort NHANES (2009–2012) [22] (Table 1). Salazar et al. (2018) also concluded that there was an inverse association between PD and higher consumption of whole-grains and fruits [32]. The protective effect of fibers against PDs could be explained because they improve glycemic control [33], which is an established risk factor for periodontitis.

Fatty acids consist of a straight alkyl chain, terminating with a carboxyl group. The number of carbons in the chain varies, and the compound may be saturated (containing no double bonds) or unsaturated (containing at least one double bond). Milk fat, coconut oil and palm oil are sources of short- and medium-chain saturated fatty acids (SFA) (4–12 carbons). Long-chain SFAs (>14 carbons) are found in other vegetable and animal fats. Many food sources are composed of different fatty acids. Olive oil contains monounsaturated fatty acids, saturated and polyunsaturated fatty acids [34]. We identified only one study evaluating the impact of total fat intake on PDs [35]. In this study, Hamasaki et al. (2017) analyzed the results of the 2005 National Health and Nutrition Survey and demonstrated a positive association between low fat intake (23.2% of the total energy) and PDs [35]. More than the total fat intake, it is the nature of the fat that must be considered [34]. The SFA intake could enhance oxidative stress, which has been shown to be associated with PDs, exerting its effects by damaging cells [36]. One other study demonstrated that omega-3 fatty acid (polyunsaturated fatty acids) has a positive impact on PDs whereas SFAs have a negative impact [37] (Table 1). Other authors focused on the omega-6 (polyunsaturated fatty acids) to omega-3 fatty acid ratio [24]. In a review, Bosma-den Boer et al. concluded that a modern, Western lifestyle, composed of refined carbohydrates and a high Omega-6 to Omega-3 fatty acid ratio promotes inflammatory processes [25]. In a randomized controlled pilot study, Woelber et al. (2017) obtained similar results concerning the correlation between a high Omega-6 to Omega-3 fatty acid ratio and PD [24]. The positive effect observed of a decrease in the Omega-6 to Omega-3 fatty acid ratio on PD [24] supports the theory of resoleomics described by Serhan et al. [38] and the related periodontal studies [39,40,41].

#### 3.1.2. Impact of Micronutrients on Periodontal Disease

Micronutrients consist of vitamins, minerals and trace elements. These are nutrients without energy value, but which are essential for chemical reactions and therefore vital to the body.

The presence of vitamins in the diet is important for maintaining periodontal health and preventing PDs [8,42] (Table 1). In a minireview, Gondivkar et al. (2018) described the association of PDs with deficiencies of vitamins A, C, E, folic acid and calcium [43]. Moreover, the antioxidant effects of vitamins can have a positive impact on the prevention and treatment of PDs [8].

Vitamin A is a group of fat-soluble compounds that are metabolically linked to all-trans retinol [44]. Animal-derived food such as milk, cheese, liver and eggs are sources of preformed vitamin A, whereas carrots, green leaves, broccoli, ripe mangos, sweet potato, orange-yellow vegetables, fruits and red palm oil are sources of provitamin A carotenoids [8,44]. According to the UK National Health Service, men aged from 19–64 years need 0.7 mg/day of vitamin A compared with 0.6 mg/day for women of the same age [44]. Several studies have concluded that vitamin A deficiency was linked to the PDs [44]. Conversely, the increase in the amount of beta-carotene (≥7.07 mg/day) consumed is linked to a decrease in the number of sites with a probing depth > 3 mm after scaling and root planing [45]. Vitamin A is thought to have a role in the maintenance of the integrity of epithelial cells [43].

The vitamin B complex family consists of thiamine (B1), riboflavin (B2), niacin (B3), pantothenic acid (B5), pyridoxine, pyridoxal, pyridoxamine (B6), biotin (B7), folic acid (B9), and cobalamin (B12). For adults 200 µg/day of vitamin B9, present in leafy greens and fortified cereals, and 1.5 µg/day of vitamin B12, present in fortified cereals, meat and fish, are recommended according to the UK National Health [44]. Few studies have analyzed the association between vitamin B and PDs. However, deficiency in vitamin B complex results in lower resistance to bacterial infections, which could explain the role of vitamin B, particularly vitamin B9, in PDs [43]. In a prospective cohort, Zong et al. analyzed the potential association between serum vitamin B12 with changes in periodontitis [46]. They demonstrated that an increase in serum vitamin B12 was associated with a decrease in the clinical parameters of PD (probing pocket depth, clinical attachment loss and, the tooth loss) [46].

Vitamin C (ascorbic acid) can be found in many fruits and vegetables. According to the UK National Health, a dose of 40 mg/day is recommended for adults [44]. Several studies have demonstrated that a low level of vitamin C was associated with PD [8,44]. In a multicenter, randomized, parallel-group, controlled clinical trial comprising 300 individuals with gingivitis, Shimabukuro et al. concluded that toothpaste with vitamin C and magnesium salt can reduce gingival inflammation [47]. Compared with conventional toothpaste, this toothpaste exhibited a significantly higher activity against reactive oxygen species, which might be associated with the onset and progression of gingival inflammation [47].

Vitamin D, which comes mainly from fortified cereals, mushrooms and fish, enhances the absorption of minerals such as calcium, iron, magnesium, phosphate and zinc [48]. It is composed of two groups, vitamin D2 (cholecalciferol) and vitamin D3 (ergocalciferol). The UK National Health recommends intake of 10 µg/day for adults [44]. Several clinical studies have demonstrated an association between a dietary vitamin D deficiency and PDs [49,50,51]. However, other studies have concluded that there is no link between the levels of serum vitamin D and periodontal health [49,52]. The link between vitamin D and PDs remains unclear and needs further investigation.

The vitamin E complex family consists of eight naturally occurring, lipid-soluble antioxidant micronutrients, four of which are tocopherols and four are tocotrienols. They are the most important lipid-soluble antioxidant and they prevent lipid peroxidation [44]. The major food sources are vegetable oils, fortified cereals, nuts, seeds, meats, fruits and vegetables [53]. The UK National Health Service recommends 4 mg/day for men and 3 mg/day for women, but in the USA the recommended daily intake is 15 mg [44,53]. Pfeiffer et al. concluded that there was no significant association between serum vitamin E levels and PDs [54]. However, in a more recent cross-sectional study including 4708 participants, an inverse association between serum alpha-tocopherols and PD severity was reported [55]. This difference could be attributed to fact that Zong et al. (2015) considered confounding factors such as blood lipids [55].

Vitamin K is a group of vitamins that plays a role in the synthesis of proteins that are precursors for blood coagulation factors, such as prothrombin and factors VII, IX, and X [56]. It also plays a role in the synthesis of proteins required for bone metabolism, such as periostin and osteocalcin. Kale, collards, spinach, and mustard are a source of vitamin K [8]. Vitamin K deficiency is thought to be associated with gingival bleeding; it was reported that vitamin K supplement failed to reduce pro-inflammatory factors in the periodontum in a rat model [57]. However, to our knowledge, no articles have studied the link between vitamin K and PD in humans.

Calcium is essential for the maintenance and formation of calcified tissues such as bone or teeth. The recommended daily intake for adults 19–64 years of age is 700 mg according to the UK National Health Service [44]. It is present in milk products, eggs, fish, leafy vegetables, seeds and nuts [8]. Several reviews conclude that a calcium-deficient diet was associated with PDs [8,44] (Table 1). In a recent cross-sectional study, Adegboye et al. demonstrated that the calcium intake was inversely correlated with PDs in Danish adults [58]. Tanaka et al., in a cross-sectional study including 1162 Japanese women with a mean age of 31.5 years, reported, after adjustment for age, smoking status, region of residence, education, household income, toothbrushing frequency and use of an interdental brush, an association between a calcium intake of >585 mg/day and a lower prevalence of PDs [59].

Magnesium, found mainly in cereals, nuts, seeds cocoa, soybeans, spinach, marine vegetables and tomatoes, is also essential for the maintenance and formation of calcified tissues such as bone or teeth, but its association with periodontal health is unclear [8,44] (Table 1). The UK National Health Service recommends daily dose for men is 300 mg/day and 270 mg/day for women [44]. In the only study identified assessing the association between magnesium and PDs, Staudte et al. recorded the 7-day food intake in 42 patients with chronic periodontitis and 38 healthy subjects and reported a negative effect of magnesium-poor diets on periodontal health [60]. However, more studies are necessary before we can conclude on the effect of magnesium intake on periodontal health.

Iron plays a crucial role in oxygen transport by hemoglobin [44]. It is also an essential cofactor for many enzymes, whose function is reduced if there is an inadequate supply of iron. The recommended daily dose of iron for men aged from 19–64 years old and for women aged from 50–64 years old 8.7 mg, and for women aged from 19 to 50 years, it is 14.8 mg according to the UK National Health Service [44]. Rich food sources of iron are fish (salmon and tuna), red meat, spinach, and dry beans [8]. In a recent study, Chakraborty et al. indicated that iron-deficiency anemia was associated with a reduction in antioxidant enzymes, and so to an increase in oxidative stress and a worsening of PDs [61]. However, no studies have studied the correlation between iron supplements and PDs (Table 1).

After iron, zinc is the most abundant trace mineral necessary for the body. The primary source of dietary zinc is protein-rich foods, spinach, nuts, fortified cereals [8,44,62]. Zinc is a cofactor in enzyme-controlled processes and it also has antioxidative properties [8,44]. Zinc starves reactive oxygen species and neutralizes bacterial toxins [63]. The UK National Health Service recommends that the daily dose for men is 9.5 mg, and 7.0 mg for women [44]. Adverse effects such as suppression of immune responses, decrease in high-density-lipoprotein cholesterol, reduction in copper absorption, and impairment of copper status have been described in case of excess intake of zinc supplements [44]. Two studies demonstrated that low serum zinc levels were associated with PDs [64,65].

Polyphenols are physiological compounds present in plants. Wine, red fruits, vegetables, grapes, green tea and coffee are food sources of polyphenols [66]. Most polyphenols have antioxidative and anti-inflammatory properties [66]. Several studies concluded on the positive effect of polyphenol in the prevention and treatment of PD. In a clinical study, it was observed in healthy patients that the use of pomegranate juice mouthwash (rich in polyphenols, tannins, ellagic acid and anthocyanins) significantly reduced the growth of *Lactobacillus* and *Streptococcus* species [67]. In PD patients, the application of a gel containing 1% curcumin (a bioactive substance of turmeric) to the affected areas in the periodontal pockets had significant bactericidal effects on *Capnocytophaga, F. nucleatum, P. intermedia* and *P. gingivalis* [68]. The effect of polyphenols has also been demonstrated on the clinical signs of PD. In a randomized clinical trial, the daily oral intake of fruit/vegetable-containing capsules reduced the pocket depth in patients with chronic PD [69]. Grover et al. (2016) demonstrated that the subgingival application of a gel containing 10% of *Emblica officinalis* or gooseberry extract reduced inflammation, pocket depth and the sulcus bleeding index [70]. In patients with PD, the use of green tea extract gel (1%) containing epigallocatechin gallate or green tea extract toothpaste with 60–90% epigallocatechin gallate decreased the clinical signs of PD [71,72].

#### 3.1.3. Impact of Probiotics and Prebiotics on Periodontal Disease

Probiotics are defined as “living microorganisms, mostly bacteria, non-pathogenic, used as nutritional supplements, which after being ingested in the right amount, improve the intestinal microbial balance and cause beneficial effects on the health of those who ingest them” [73]. Some probiotics are thought to have a function in the maintenance of periodontal health and the treatment of PD. In a review, Matsubara et al. analyzed 12 RCTs and concluded that oral administration of Lactobacillus reuteri (DSM17938, ATCC 55,730 + ATCC PTA5289, 1 × 10^8^ CFU), Lactobacillus salivarius (WB21, 6.7 × 10^8^ CFU) and Lactobacillus brevis (CD2, 0.2 × 10^8^ CFU) had a positive impact on the clinical signs of PD and reduced the levels of major periodontal pathogens; however, the benefits were maintained only with continuous probiotic administration [74]. Morales et al. raised the same conclusions for the same strains and also concluded on the Lactobacillus plantarum (L137) in supportive periodontal therapy [75]. Several mechanism could explain the action of probiotics: (i) production of lactic acid that inhibits the proliferation of periodontal bacteria by penetrating the membrane of bacteria and acidifying the cytoplasm, (ii) production of hydrogen peroxide that inhibits the growth of pathogenic bacteria, (iii) modification of proteins at the level of the site of attachment, (iv) production of inhibitory substances such as bacteriocins, (v) production of vitamins or other substances [75].

Prebiotics are “non-digestible food ingredients that beneficially affect the host by selectively stimulating the growth and/or activity of one or a limited number of bacteria in the colon, and thus improves host health” [76]. In the only study selected for this review, Chandra et al. (2016), reported the efficacy of a probiotic, Saccharomyces boulardii (1.6 × 10^9^ CFU), mixed in a ratio 4:1 with a prebiotic, fructooligosaccharide, in the treatment of PD when used as an adjunct to non-surgical periodontal therapy [77].

Thus, our review of the literature demonstrated that nutrition has an essential role in the development of PD (Figure 3). Particularly, the composition of the diet in terms of the main macronutrients and micronutrients was responsible for the onset of PDs. There is insufficient evidence of an association between prebiotics and probiotics and PDs.

### 3.2. Nutrition as a Risk Factor for Chronic Diseases

Human nutrition varies according to cultural, religious, and ideological practices, and it also can be modified by health concerns. Among the many diets around the world, some have been reported to have an impact on CDs. The Mediterranean diet, the Okinawa diet, the Dietary Approaches to Stop Hypertension (DASH) and the Vegetarian diet are reported to have a positive impact on CDs such as CVDs, diabetes and cancers, whereas the Western diet is reported to be an unhealthy diet [78].

#### 3.2.1. Unhealthy Diets: Western Diets

Western diets have high calorie count and are high in proteins, fatty acids and sugar [79]. They consist mainly of meat, industrialized food, sugar, refined grains, alcohol, salt and fructose, with an associated reduced consumption of fruits and vegetables [79]. Many people in the United States and many other Western countries have adopted this diet. The evidence shows that a diet high in saturated fat and sucrose increases the risk of several chronic conditions including CVD, diabetes and cancers, compared with a diet of low-energy foods, such as fruits and vegetables [80].

In a meta-analysis of 22 cohort studies Rodríguez-Monforte et al., (2015) reported in a comparison of the highest and the lowest category of Western diets, the pooled relative risk for CVD, stroke and coronary heart disease was 1.14 (95% CI, 0.92–1.42), 1.05 (95% CI 0.91–1.22), and 1.03 (95% CI 0.90–1.17), respectively [81]. However, other observational studies and reviews concluded to a significant increased risk in case of PD [82,83]. In a cohort study, Oikonomou et al. (2018), observed that the Western dietary pattern was predictive of severe coronary artery disease (AUC: 0.73, 95% CI, 0.64–0.83, *p* < 0.001). The high consumption of ultra-processed foods—commonly high in one or more of the following element: calories, saturated fat, sugar or sodium—seems to be one key element of the association between the Western diet and the higher risk of cardiovascular, cerebrovascular, and coronary heart diseases [84]. The salt content in these diets could be another key element because WHO estimated that a decrease in salt intake from the current global levels (9–12 gm/day) to the recommended level (5 gm/day) would have a major impact on reducing blood pressure and the incidence of CVDs [85]. In a study including 31,672 subjects, He et al. (2014) observed that a decrease of 1.9 g of 24 h urinary sodium was associated with a reduction in 42% of stroke deaths and 40% of coronary heart disease deaths [86]. Additionally, the Western diet contains additives, such as sweeteners, emulsifiers, thickeners, preservatives and food colorings, some of which have already been associated with CD, such as coronary diseases [79].

In a matched case-control study, Beigrezaei et al., (2019) observed that Western diet significantly increased the risk of type-2 diabetes (OR: 9.25, 95% CI, 4.95–17.4) [87]. Nagao et al. observed the same correlation [88]. Meat in the Western diets could be responsible due to their composition in high saturated fatty acid content that are responsible for obesity, hyperinsulinemia and hyperglycemia. The higher sugar intake observed in Western diets is also associated with the increasing risk of type 2 diabetes [89].

Western diets were reported to have a negative impact on the risk of colorectal, stomach and prostate cancers but not on the risk of breast, esophagus and rectal cancers [80,90,91,92,93,94,95,96,97,98]. The consumption of high quantities of meat in the Western diet could be one of the key elements of its impact on cancer. The International Agency for Research on Cancer has classified red meat and processed meat as “probably carcinogenic to humans’ and ‘carcinogenic to humans”, respectively [99]. In an observational study including 104,980 participants, Fiolet et al. (2018) demonstrated that the overall risk of cancer was increased by 10% if the proportion of ultra-processed foods was increased by 10% [100]. However, results from a meta-analysis failed to show a positive correlation between red and processed meat, cooking methods, and the concentration of heterocyclic amines with the risk of prostate cancer [101]. Thus, Western diets may have a more complex impact on cancer risk than the impact from processed and red meat as individual aliments.

#### 3.2.2. Healthy Diets: Mediterranean Diet, Okinawa Diet, Dietary Approaches to Stop Hypertension and Vegetarian Diet

- Impact of the Mediterranean diet on CDs

The Mediterranean diet is associated with a high intake of extra-virgin olive oil, fruits, vegetables, nuts, cereals and legumes, moderate intakes of fish, and low intakes of red meat, sweets and eggs [102]. This diet is rich in polyphenols, lycopene, trace elements, minerals, vitamins and omega-3 fatty acids [103]. Studies conclude that the Mediterranean diet, known as a “long life” diet, even when it is adapted to other countries’ eating habits, has a key role in health promotion and CD prevention [104,105]. In an umbrella review of meta-analyses of cohort studies, Galbete et al. reported a 19–27% lower risk of developing CVDs (e.g., coronary heart disease, stroke), a 13–23% lower risk of developing type-2 diabetes and a 14% lower risk of cancer mortality in subjects adhering to the Mediterranean diet compared with those with the lowest adherence levels to the diet [106].

In an observational prospective population-based cohort study including 23,232 men and women, Paterson et al. (2018) reported that subjects adhering to a Mediterranean diet had a lower risk of stroke [107]. In an observational study including 1284 patients hospitalized for an acute or chronic ischemic heart disease Siki et al. (2017) reported similar reductions in risk [108]. Based on the analysis of data from the National Nutrition and Physical Activity Survey, including 9435 subjects, Aridi et al. reported that higher adherence to a Mediterranean diet decreased the incidence of CDs, with lower concentrations of total cholesterol, low-density lipoprotein, diastolic blood pressure and dyslipidemia [109]. One key component of this diet is the olive oil that acts on the risk of CVDs by (i) reducing the concentrations of low-density lipoproteins, (ii) increasing the concentration of high-density lipoproteins [110] and, (ii) downregulating pro-inflammatory cytokines such as interleukin-6 and tumor necrosis factor [111].

The positive impact of the Mediterranean diet on type-2 diabetes was also observed in several studies [112,113,114]. The results from the PREDIMED (PREvención con DIeta MEDiterránea) randomized trial, showed a reduction of 40% in the incidence of type-2 diabetes in subjects adopting the Mediterranean diet with extra-virgin olive oil but without energy restriction compared with subjects adopting a low-fat control diet [115]. In a recent review Martín-Peláez et al. analyzed clinical trials and prospective cohort studies including more than 100,000 participants [116]. They reported similar results and underlined that the positive impact is due to olive oil, which helps glycemic control by lowering fasting plasma glucose and HbA1c. The preventive action of the diet is probably due to the anti-inflammatory and antioxidative properties of its components. The diet is thought to act by improving autophagy, Th cells imbalance, cell adhesion, complement activity and oxidative stress [103].

The Mediterranean diet also was reported to have a positive impact on the risk of cancer [117,118,119,120]. For example, in a European cohort, with 5,296,617 person-years of follow-up, Bamia et al. observed an 8–11% decreased risk of colorectal cancer in subjects adhering to the diet [121]. In a multicenter case-control study, high adherence to a Mediterranean diet was associated with an almost 35% reduction in risk of bladder cancer compared with low adherence [122]. It is thought that the components of the diet reduce the cancer risk by reducing tumor cell growth through anti-oxidative and anti-inflammatory effects (vegetables, fruit, and olive oil), increasing chemoprotective effects (olive oil), and inhibiting tumor development (dairy products) [123,124,125,126];

- Impact of other Mediterranean-type diets on chronic diseases

The Okinawa and Dietary Approaches to Stop Hypertension (DASH) diets have a similar composition to that of Mediterranean diets [127].

The DASH diet is rich in vegetables and fruits, whole grains, low-fat dairy products, poultry, fish, nuts, beans and seeds. Compared with a Western diet it, contains less sugar, sodium, fats and red meat. This diet is rich in nutrients (protein, magnesium, calcium, potassium and fiber) and poor in trans-fatty acids, saturated acids and cholesterol. In a literature review of prospective cohort studies, Chiavaroli et al. (2019) reported that the DASH diet was associated with a decrease in the incidence of CVD (RR = 0.80; 95% CI, 0.76–0.85), coronary heart disease (RR = 0.79; 95% CI, 0.71–0.88), stroke (RR = 0.81; 95% CI, 0.72–0.92), and diabetes (RR = 0.82; 95% CI, 0.74–0.92) [128]. Mohsenpour et al. (2019) reported that high adherence to a DASH diet plan was associated with a decrease in any cancer mortality (RR = 0.84; 95% CI, 0.81–0.86) [129]. Several systematic reviews, including randomized controlled trials and observational prospective studies, reported the association of the DASH diet plan with lower risks for CVDs, coronary heart disease, stroke, heart failure, diabetes, and several types of cancer [130,131,132,133,134,135,136].

The Okinawa diet is rich in vegetables (green, yellow and roots) and fibers, has a moderate fish intake and low calorie, meat, dairy product, fat, and carbohydrate intake. In contrast with the traditional Japanese diet, the traditional Okinawa diet consists of smaller quantities of rice, replaced by sweet potato [78,137]. The Okinawan diet has only 30% of the average Japanese sugar intake, and this is mainly from fruit [78]. However, the Okinawa diet has disadvantages, including high sodium intake (soy sauce, miso soup), and unnecessary restrictions of specific food groups (e.g., meat, eggs) [78,137]. Although the Okinawa diet has been reported to have a positive impacts on the prevention of CDs, not many studies have been published in the last 10 years [78,137,138].

- Impact of vegetarian diet on chronic diseases

The vegetarian diet is a mode of nutrition based on the exclusion of all meat poultry and fish. Vegetarian diets are classified in different subgroup: vegetarian diet (no animal meat, but consumes eggs and milk), semi-vegetarian (occasional meat consumption), lacto-ovo-pesco-vegetarian (consumes fish) and vegan (no animal-derived products) [139]. In general, vegetarian diets are associated with health benefits and the results from some studies show that vegetarians tended to have a better quality diet than meat-eaters and a higher intake of important nutrients like fiber, vitamin C, vitamin E and magnesium [140].

The adherence to a vegetarian diet was shown to be associated with a reduction in CVD mortality [141]. In a meta-analysis including seven studies and 12,4706 subjects, Huang et al. (2012) reported a significant lower mortality due to coronary disease in vegetarians (RR = 0.71; 95% CI, 0.56–0.87) compared with non-vegetarians [142]. Similar results were reported by Kwok et al., (2014) and Dinu et al., (2017) [143,144]. The vegetarian diet has a positive impact on CVD risk factors, such as blood pressure, blood lipids, platelet aggregation, obesity, and metabolic syndrome [141,145,146]. The vegetarian diet has a role in the prevention of CVDs as well as in its treatment [141].

An important impact of the vegetarian diet was demonstrated in the prevention and treatment of diabetes [147,148]. In an observational study including 2918 subjects, Chiu et al., (2018) found that the adherence to a vegetarian diet for 5 years reduced the incidence of diabetes by 35%. A decrease of 53% was observed for subjects adhering to a vegetarian diet after having been non-vegetarian [149]. In a randomized controlled trial including 74 subjects, Kahleova et al. (2011) concluded that 43% of subjects adhering to a vegetarian diet were able to reduce the use of diabetes medication to treat compared with non-vegetarians [150]. A systematic review that included six studies reported an association of vegetarian diet and improved blood sugar control in people with type 2 diabetes [145].

The vegetarian diet seems to decrease the overall cancer risk [151]. In a case-control study including 469 subjects, Chang et al. (2017) observed a lower risk of breast cancer in vegetarians [152]. Similar results were obtained by Tantamango-Bartley et al. (2013) [153]. A vegetarian diet decreased the risk of cancers of the gastrointestinal tract (HR = 0.76; 95% CI, 0.63–0.90) [153]. A vegan diet reduced the overall incidence of cancer (HR = 0.84; 95% CI, 0.72–0.99) and the risk of prostate cancer (HR = 0.65; 95% CI: 0.49–0.85) [153,154]. A lacto-ovo-vegetarian diet had a positive impact on the risk of gastrointestinal cancers (HR = 0.75; 95% CI, 0.60–0.92) [153]. A pesco-vegetarian diet reduced the risk of colorectal cancer [155]. In contrast to these results, a review by Molina-Montes et al. (2020) explained that due to the low number of studies available (five studies included), it was not possible to conclude that a vegetarian diet had a preventive role on the overall incidence of cancer [151].

Although there is evidence that vegetarian diets can prevent CDs, it is important to acknowledge that vegetarian diets have some risks. This diet is rich in linoleic acid (LA) and poor in alpha-linolenic acid (ALA), with a higher LA to ALA ratio than the recommended 4:1 ratio [156]. In 2020, Fallon et al. highlighted that vegetarian and vegan diets could be associated with a lower intake of vitamins (B12 and D), calcium, iodine and selenium in UK adult women [157]. These micronutrients play a key role in fetal development, and low intake results in a negative impact on CDs. In the long-term, vegetarian diets low in legumes, nuts and/or dairy and eggs could have a negative impact on health due to severe protein deficiencies which could increase risk muscle wasting or atrophy [158], or Kwashiorkor disease in cases of extreme severe deficiency, although there are not many publications about this [159].

The results from our review demonstrated that the diet could have an adverse or positive impact on CDs. It confirmed a strong association between nutrition and CVDs and nutrition and diabetes but only a more moderate association between nutrition and cancer, which was dependent on the type of cancer (Figure 3). The diet pattern and the nutrients have an impact on CD risk. The Nutrition and CDs Expert group reported that fruit, vegetables, beans/legumes, seafood, fish, nuts/seeds, yogurt, fiber, whole grains, omega-3, polyunsaturated fats and potassium all had protective cardiometabolic effects, and processed and unprocessed red meats, beverages, trans-fats, glycemic load and sodium all had negative cardiometabolic effects [160].

### 3.3. Periodontal Diseases as Risk Factors of Other Main Chronic Diseases

#### 3.3.1. Association between Periodontitis and the Main Chronic Diseases

PDs have been reported to be associated with CDs such as CVD, diabetes, respiratory diseases, rheumatoid arthritis, cancers, Alzheimer disease [10,161,162]. In our review, we focused on the main CDs (cardiovascular diseases, diabetes and cancers).

PDs have been reported to be associated with several CVDs, such as peripheral arterial disease, stroke, and ischemic heart disease [10,163,164]. Statistically significant associations were found between CVD and gingivitis (OR = 4.30; 95% CI, 1.85–10.00), and CVD and periodontitis (OR = 2.87; 95% CI, 1.04–7.93) [165]. In a literature review of 22 observational studies, Xu et al. (2017) observed an increased risk of myocardial infarction associated with PDs (OR = 2.02; 95% CI, 1.59–2.57) [166]. These data were confirmed by several other articles [167,168,169]. The link between PD and stroke was reported in several reviews [170,171]. Leira et al. (2017) reported a relative risk of 2.52 (95% CI, 1.77–3.58) for pooled cohort studies and 3.04 (95% CI, 1.10–8.43) for pooled case-control studies [170]. PDs were also reported to be associated with coronary heart disease [172]. Although the association between PD and CVDs has been reported, the causal association is not clear as PDs and CVDs exhibit multifactorial etiologies and share a common risk. However, periodontal bacteria seem to have a key role since biopsies of 17 coronary arteries in patients with atherosclerosis revealed that periodontal pathogens was present in approximately 60% of samples: *P. gingivalis* was present in 52.9%, *A. actinomycetemcomitans,* in 35.5%, *Prevotella intermedia,* in 23.5%, and *Tannerella forsythia,* in 11.7% [173].

Analyses of the National Health and Nutrition Examination Survey (2009–2010, 1165 diabetes-free adults) showed that the PD was positively associated with prevalent impaired glucose tolerance, a sign of prediabetes [174]. This association could be explained by the fact that PDs could alter glycemic control by increasing the resistance of tissues to insulin [175]. The treatment of PDs can improve glycemic control supporting this explanation [176,177,178,179,180]. The dysbiosis of oral microbiota could explain the association between PDs and diabetes because the subgingival microbial profile, particularly for three bacteria (*Tannerella forthysia, P. gingivalis and A. actinomycetemcomitans*), are different in patients with and without diabetes [181]. The concentrations of *P. gingivalis* were significantly higher in patients with uncontrolled diabetes compared with those with controlled diabetes [182].

A positive correlation between PDs and pancreas, head, neck and lung cancers has been reported [162]. Corbella et al. (2018) reported a trend of increased cancer risk in patients with PDs but could not conclude because of the small number of studies [183]. Güven et al. (2019) reported a negative association between PDs and cancers in an observational study, including 5199 patients [184]. With a follow-up of 7.2 years, patients with PDs had a 17% higher risk of cancer compared with those without PDs, for the same age groups and gender. PDs increased the risk of pancreas cancer (RR = 1.74; 95% CI, 1.41–2.15) [185]. Similar results were obtained for lung cancer (HR = 1.24; 95% CI, 1.13–1.36) [186], breast cancer (RR = 1.22; 95% CI, 1.06–1.40) [187], oral cancers (OR = 3.21; 95% CI, 2.25–4.16) [188], head and neck cancer (OR = 2.63; 95% CI, 1.1.68–4.14) [189]. Periodontal pathogens could be responsible for the association between PDs and cancer. Some periodontal bacteria have been observed in the organs affected by cancer. For example, *P. gingivalis* was found at significantly elevated levels in patients with oral squamous cell carcinoma [190] and squamous cell carcinoma of the esophagus [191]. The presence of *Fusobacterium nucleatum* was higher in human colon adenomas, compared with the surrounding tissues, and in stool samples from patients with colorectal cancer, compared with those without colorectal cancer [192].

The evidence from this review supports PDs as risk factors for CVDs, type-2 diabetes and cancers. The presence of oral bacteria, particularly periodontal bacteria, could be responsible for this association [10,193,194].

#### 3.3.2. Periodontal Bacteria as the Link between Periodontal Diseases and Chronic Diseases

The main factors responsible for PDs are the presence of periodontal pathogens, such as *P. gingivalis*, *Treponema denticola* and *Tannerella forsythia* in the subgingival microbiota [195]. These periodontal bacteria induce a dysbiosis of the microbiota, and the resulting inflammatory process leads to PD. These bacteria, their bacterial products and the inflammatory molecules produced in response to the presence of these bacteria can circulate, via the blood and the digestive pathways, to attain different organs [10,196,197]. The presence of oral bacteria in various organs involved in systemic pathologies has been reported in patients suffering from PD [196,197]:

- Diffusion of bacteria via blood

Bacterial penetration from the oral cavity into the underlying tissues and the blood system is only possible if one of the protective systems is defective. This failure can take place at the level of (i) the physical protection provided by oral epithelium which contains antimicrobial peptides, (ii) the electrical protection, due to the difference between the host cell and the bacterial layer, (iii) immunological protection by antibody-producing cells, (iv) phagocytic protection by the reticulo-endothelial system [196]. Patients with PDs have a high risk of bacteremia due to the increasing number of periodontal bacteria [196]. Generally, the microorganisms, which are mainly anaerobic bacteria, will be eliminated by the reticuloendothelial system within a few minutes and do not cause other clinical symptoms except for a possible slight increase in body temperature [196]. However, in some cases, the disseminated microorganisms will settle in a site where conditions are favorable for their growth; thus, bacteremia can lead to cardiovascular CDs or diabetes [10,198]. Alternatively, the production or the release of toxins such as exotoxins, endotoxins, bacterial proteins can enter into the bloodstream and, thus, be disseminated throughout the body, attaining organs where they cause CDs [10,197]. Finally, oral bacteria can induce local inflammation and the inflammatory molecules produced can diffuse in the body through the bloodstream and provoke acute and chronic inflammatory reactions at a distance from the infection site. Bacteria can also enter the bloodstream, react with specific circulating antibodies, and form macromolecular complexes that can give rise to a variety of acute and chronic inflammatory reactions at deposition sites [10,198]. The treatment of PDs leads to both a reduction in the systemic inflammatory burden and an improvement in endothelial function [199].

- Diffusion of bacteria through the digestive pathway

Although the mechanism is unknown oral bacteria, bacterial products and inflammatory molecules can also diffuse through the digestive tract, but they must be able to resist stomach acidity to survive and multiply in the gastrointestinal tract [200,201]. While the link between bacterial colonization of the intestine, intestinal inflammation and CDs has not been established, results from an observational study showed that oral bacteria can colonize and persist in the intestines, leading to activation of the immune system and chronic inflammation [202]. Oral bacteria can invade the intestinal tract directly through the esophagus, provoking intestinal dysbiosis and affecting the digestive system, leading to systemic disease [10]. Thus, several studies have reported that the passage of *P. gingivalis* through the intestine via the digestive tract is implicated in many diseases, such as colon cancer, chronic inflammatory bowel disease and diabetes. *P. gingivalis* induces dysbiosis by altering the innate defenses of the host and promoting inflammatory responses in phagocytic cells [203].

## 4. The Association between Nutrition, Periodontal Disease and Chronic Diseases

Our study has some limitations due to the choice of the review for developing and presenting summaries of evidence. Decisions about attributing a GRADE level are subjective but, to minimize bias, each article was assessed by two authors and we observed little disaccord between the GRADE levels attributed. We also excluded beverages (coffee, sodas, alcohol) that are modifiable risk factors, to focus the findings on nutriments and nutritional intake.

Nevertheless, this review provides strong evidence that nutrition is a common modifiable risk factor for PDs and other CDs. High-sugar, high-saturated-fat, low-polyols, low-fiber and low-polyunsaturated-fat intake cause an increased risk of PDs. This pattern of nutrients is classically found in the Western diet, which is considered as an ‘unhealthy’ diet that causes CVDs, diabetes and cancers. Conversely, low sugar, high fiber and high omega-6 to omega-3 fatty acid ratio intake reduces the risk of PDs. The Mediterranean, DASH, vegetarian and Okinawa diets that correspond to these nutritional intakes are considered as ‘healthy’ diets, and reduce this risk of CVDs, diabetes and cancers. The role of micronutrients, such as vitamin D, E, K and magnesium, remains unclear, while others, such as vitamin A, B, C, calcium, zinc and polyphenol, have been shown to prevent PDs. Hence, Western diets that have a low intake of these latter nutriments cause PDs and Mediterranean diets, with a higher intake, lead to periodontal health [24,204]. These results are observed in specific populations. The adoption of the Mediterranean diet in a group of overweight/obese subjects led to a significant decrease in periodontal pathogens such as *P. gingivalis*, *P. intermedia* and *T. denticola* in the saliva [205]. The Okinawan-based nordic diet improved the general and periodontal health in diabetic patients [206]. Some evidence suggests that probiotics and prebiotics could promote good periodontal health, but more studies are required.

A bidirectional association seems to exist between nutrition/PDs and nutrition/CDs and although it has very rarely been assessed, it seems that PD or CDs could impact the nutrition. Sheiham et al. showed that a lower intake of vitamins, calcium, essential fatty acids, protein, and total calories was significantly associated with lower numbers of natural teeth [207]. An adult population with coronary artery disease and PDs were reported to have a significantly higher percentage of their total energy contribution from beans, fried food and sweets, and to eat less fruit than people without PDs [208]. Longitudinal studies with representative samples are needed to confirm these results. The studies assessing associations between PDs and nutrition have essentially focused on the potential consequences of PDs on adults’ quality of life. PDs can result in clinical signs and symptoms, including tooth mobility, bleeding, gingival recession and dental loss, which have a negative impact on daily life [209]. Since PDs are a major cause of tooth loss in adults, people with PDs are at risk of multi-tooth loss, edentulism and chewing dysfunction, which will affect their nutrition, quality of life and self-esteem [210]. Poor oral health can modify food choices negatively, which could lead to sub-optimal nutritional condition. Hence, it is important to identify and treat periodontal and dietary problems to improve quality of life and health [43]. It has been shown that it is easier to raise awareness of the need to change eating habits in an individual with CD than in healthy people. Thus, secondary prevention has a better influence on dietary behavior than primary prevention [105]. Very few studies have analyzed the influence of CDs on dietary behaviour. In an observational study, Chong et al. (2017) concluded that the diagnosis of diabetes did not significantly change patients’ diets [211]. In contrast, patients experience a reduction in their quality of life during and after cancer treatment, and due to several symptoms, these patients modify their diets [212]. However, more studies are needed to confirm these results.

**Figure 4:**
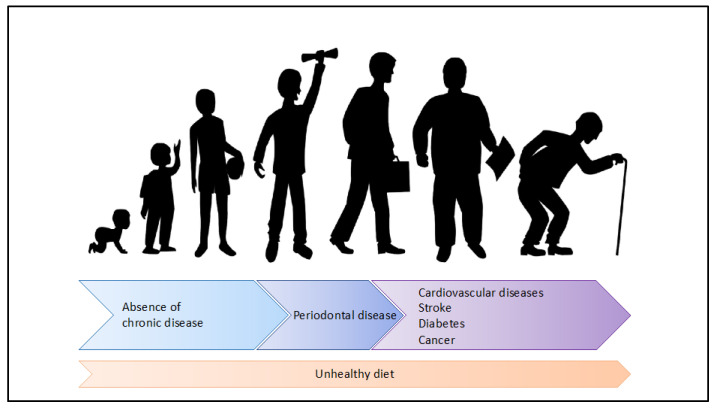
Nutrition as key risk factor of the periodontal disease and other chronic diseases during the life course.

Although PDs, CVDs, diabetes and cancer are all considered as CDs, they occur at different ages. World Health Organization, reported a very high prevalence of PDs in individuals aged 35 to 44 years old in 35 countries [14]. Periodontal bacteria and interdental inflammation has been reported in people aged 20 to 35 years old [213,214]. In contrast, the risk of CVD, diabetes and cancers increases with age. The risk of CVD is higher in men aged 55 years and women aged 65 [215]. The risk of diabetes is higher after 45 years old [216]. The risk of cancers starts to increase after 40 years of age but is highest at 65–69 years [217]. Therefore, over the life course, depending on age, a person will have a different risk of developing a CD. At first, the person will be at risk of developing periodontal disease and then, as age advances, he will be confronted with other chronic diseases such as CVDs, diabetes or cancer (Figure 4).

Since nutrition is a risk factor for PDs and other non-communicable diseases and the risk for PDs appears early in life, it seems likely that nutritional control is the key factor for prevention because it can prevent PDs and, therefore, other main CDs. In addition, a healthy diet can contribute to the treatment of PDs and, consequently, this can help to prevent the apparition of CDs.

The results of this scoping review have implications for prevention and health promotion. First, nutritional deficiencies at certain stages of life can have short- and long-term consequences, including intergenerational effects [218]. Considering nutrition throughout life also requires an integrated offer of health and nutrition services by health care systems in all contexts [219]. Secondly, three of the most prominent noncommunicable diseases—cardiovascular disease, cancer, and diabetes—are linked by common preventable risk factors related to unhealthy diet. Therefore, action to prevent these diseases should focus on controlling the risk factors in an integrated manner. Several evidence-informed actions exist to address nutrition actions relevant to adolescents and adults, i.e., promotion of health across the life course and prevention and control of CDs based on an integrated risk factor approach [220]. Thirdly, a dedicated effort is required to mobilize collective action for health. PD control and public health need to take integrated approaches to health promotion and disease prevention based on common risk factors [219]. At the least, interprofessional education should be an important part of the modern educational landscape. Introducing an education program for health practitioner students can provide them with adequate knowledge, awareness, confidence, and attitude regarding oral and chronic health issues in link with the in connection with the essential nutrition knowledge [221]. It can help them in changing behavior, prevention and ongoing CDs surveillance. Therefore, future dentists, one of the largest health professions, providing the initial contact with patients in many cases, would be put in a position where they can significantly help reduce oral and chronic health diseases.

The clinical relevance of the results of these studies highlight that nutrition is a key component of PD prevention. Health care professionals should teach their patients how to adopt a healthy diet. According to our review, to prevent the onset of PD and, later, CDs, the diet should be low in sugar and saturated fatty acid but rich in polyols, fibers, polyunsaturated acids, vitamin A, vitamin B, vitamin C, calcium and polyphenols. At the same time, they must also teach oral hygiene practices because oral hygiene is another key component of PD prevention [14]. Professional preventive measures must be based on appropriate periodontal diagnosis, as mechanical plaque removal alone is inappropriate as treatment of PD [222]. Oral hygiene should be taught very early in life because it has been shown that young adults already have a dysbiotic oral microbiota and signs of interdental inflammation that may progress to PD [213,214]. Priority should be given to the disorganization of oral biofilm, especially in the interdental spaces, as part of an individual oral prophylaxis that is delivered and controlled for each patient [223]. Prevention and control of the inflammation gingival process is a critical component for the prevention of PD. Behavioral change and risk factor control approaches should be incorporated in preventive efforts. Professional measures, including actions for themselves and for persons under their care, should be used to prevent disease and maintain quality of life. With appropriate diet and nutrition, primary prevention of many PDs and, consequently, CDs can be achieved.

While there are a number of evidence-based actions to address nutrition actions in relation to periodontal health and relevant CD prevention, the available evidence is limited in some knowledge areas. Further research could be merited in the listed areas: (i) Determining the daily quantity of each nutrient associated with periodontal health and general health; (ii) Increasing the level of evidence for the coexistence of diet with related CD risk factors in adolescence and adults. If an incorrect nutritional intake correlates to a state of oral disease, the inverse relationship is not proved. Compromised oral health can alter food choices and negatively impact food intake leading to suboptimal nutritional status which can lead to CDs [43]; (iii) Promoting the comprehensive integrated assessment, diagnosis, prevention and management of all forms of malnutrition on the prevention and management of PD [224]; (iv) Establishing follow-up action implementation research to help identify delivery innovations that reach and affect populations in order to achieve scale-up, health systems integration and sustainability. In particular, early interactions between different health professionals could help to understand the overall issues and the importance of interpersonal skills in the healthcare workplace. This will address the concern of low knowledge among physicians regarding oral health, the relationship between PD and systemic health.

## 5. Conclusions

In conclusion, this review gathers evidence for the potential relationship between nutrition/PDs, nutrition/CDs and PDs/CDs, providing a detailed review of key resources for healthy and nutritional habits. More than healthy diet habits, we synthetized the micro- and macro-nutrients that are beneficial for health. This highlights that, during his life course, an individual adopting an unhealthy diet will be at risk of periodontal disease. Later, if his eating habits remain unchanged and the PD untreated, then he will be at risk of other CDs, such as neuro- and cardio-vascular diseases, cancers and diabetes. Therefore, the PD could thus be considered as an early risk factor of the appearance of the other chronic diseases, a kind of sentry disease. In the complex multi-component setting composed of nutrition/PD/CDs, the main lesson to be taken home is the importance of common, integrated objectives and strategies for the prevention of chronic diseases, of which nutrition is the cornerstone.

## Figures and Tables

**Table 1:** Nutrients and their rule in the risk of periodontal disease.

Nutrients		Risk of Periodontal Disease ^1^
Macronutrients		
Carbohydrates	Sugars	↑ (●●●●)
	Polyols	↓ (●●●●)
	Fibers	↓ (●●●●)
Fat	Saturated fatty acids	↑ (●●●●)
	Polyunsaturated fatty acids	↓ (●●●●)
Micronutrients		
Vitamins	Vitamin A	↓ (●●●●)
	Vitamin B	↓ (●●●○)
	Vitamin C	↓ (●●●●)
	Vitamin D	↓ (●○○○)
	Vitamin E	↓ (●○○○)
	Vitamin K	Not determined
Calcium		↓ (●●●○)
Magnesium		↓ (●○○○)
Iron		↓ (●○○○)
Zinc		↓ (●●○○)
Polyphenols		↓ (●●●●)
Prebiotics and probiotics		↓ (●○○○)

**^1^** Increased risk of periodontal disease is indicated by the symbol ↑ and decreased risk of periodontal disease is indicated by the symbol ↓. The level of evidence was codified: high (●●●●), moderate (●●●○), low (●●○○) and very low (●○○○).

## Data Availability

No new data were created or analyzed in this study. Data sharing is not applicable to this article.
